# Publication practice in Taxonomy: Global inequalities and potential bias against negative results

**DOI:** 10.1371/journal.pone.0269246

**Published:** 2022-06-01

**Authors:** Rodrigo Brincalepe Salvador, Daniel Caracanhas Cavallari, Douglas Rands, Barbara Mizumo Tomotani

**Affiliations:** 1 Museum of New Zealand Te Papa Tongarewa, Wellington, New Zealand; 2 Faculdade de Filosofia, Departamento de Biologia, Ciências e Letras de Ribeirão Preto, Universidade de São Paulo, Ribeirão Preto, SP, Brazil; 3 Victoria University of Wellington, School of Biological Sciences, Wellington, New Zealand; 4 Animal Ecology Department, Netherlands Institute of Ecology (NIOO-KNAW), Wageningen, Gelderland, The Netherlands; Sao Paulo State University (UNESP/FCL/Assis), BRAZIL

## Abstract

There is broad recognition by practicing taxonomists that the field is going through a crisis, which has been dubbed the “taxonomic impediment”. There are many aspects involved in said crisis, but publication practices in taxonomy are often neglected or relegated to the backseat. We provide an initial foray into this topic via a worldwide survey with taxonomists, spanning all botanical and zoological groups, and career stages. Demographically, most of the respondents identified themselves as males (70%), working in Europe or North America (68%), in universities (50%) or museums (27%). Over half of the respondents are established/late-career researchers (only about 25% of full professors were female), with a low number of early-career researchers and graduate students (i.e., taxonomists in training). Nearly 61% of the men acquired their highest title at least eleven years ago, while only 41% of the women did so. Nearly 92% of the respondents have published new species descriptions, while around 60% and 26% have synonymized, respectively, species-level or subspecies-level taxa. In general, respondents perceive the act of describing new species to be easier than synonymizing species (*p* = 0.05). Established/late-career researchers and male researchers, particularly in Oceania and North America, found it easier to publish nomenclatural acts such as new species descriptions, while early-career researchers had their acts contested more often. Our results reaffirm the low academic recognition of the field, the lack of funding for research and publishing charges especially in the Global South, and the difficulty in finding specialized outlets (and the low impact factor of those journals) as persistent issues in taxonomy. Other significant problems raised by respondents include ethical issues in the peer-review process, a bias against newcomers in the field coming either from established researchers or committees, and taxonomic vandalism.

## Introduction

Broadly speaking, Taxonomy is the scientific discipline that circumscribes, names, and classifies living organisms [1]. As such, it forms the basis for all other endeavours in the life sciences. However, despite its central importance, taxonomy is assailed by several ills: lack of glamour; pointless bureaucracy in securing permits; lack of interest, driven mainly by the low impact factors of taxonomy journals (a result of the non-citation of taxonomic works by researchers in other areas); lack of funding; consequent lack of interested students to carry the torch; and the struggle of working against the clock in face of the present biodiversity crisis [1–6]. The crisis in this field of research has been dubbed the “taxonomic impediment” and if one talks to any taxonomist for five minutes, they will list all these issues and give a plethora of examples (the “whining about the state of taxonomy” *sensu* [7]).

However, one issue that usually takes a prominent place is publishing taxonomic works, a fact that is, in all likelihood, linked to the ‘publish or perish’ culture [e.g., 8–10]. The present project arose from discussions with colleagues, located in different countries and specializing in different taxa, regarding their experience and the difficulties they face in publishing taxonomic works. Some colleagues felt that publishing new species was getting harder due to an apparent request for more types of data (fine microscopy, DNA sequences, etc.). Others, however, felt that publishing new species was too easy while publishing revisions and synonymization of taxa was becoming less fashionable and required much more data and analyses than publishing a new taxon. The latter opinion, in particular, would be extremely problematic if true, because it would signify a bias against the publication of “negative” results. In this sense, synonymizing taxa (as opposed to describing new ones) could be understood as analogous to the typical negative results of experimental research. Bias against negative results is a widespread ill in academia, with serious consequences [e.g., 11–14]).

Given that we perceived such disparity among our colleagues’ perceptions regarding publication practices in taxonomy, we decided to investigate this matter further by presenting a survey to taxonomists worldwide. We gathered data from taxonomists working across all botanical and zoological groups, in different career stages and countries. We inquired about their perceptions and personal experience when publishing taxonomic works and present our findings below.

## Material and methods

The survey was created and presented on Google Forms (Google Inc.), counting with an introductory statement explaining the survey and acquiring written consent upon participation, followed by 15 questions (see Appendix). The questions were arranged as follows: the first eight questions (Q1–Q8) pertain to personal information, demographics, and academic background and expertise; the following six questions (Q9–Q14) pertain to taxonomic research and publication, including an open-ended question (Q14, non-mandatory) for more specific comments the respondents might want to make; the final question (Q15, non-mandatory) asked whether respondents wanted to be informed of our results. Given the nature of the subject and our goals, different types of questions (e.g., open- vs close-ended, single vs multiple-choice, rating scale) were used depending on the circumstance (see S1 Appendix).

The survey was completely anonymous; however, in Q15 respondents were given the choice to provide their email address if they wanted to hear back from us with updates on the research and eventually, our final results. No sensitive information was requested and, in all questions involving personal information, participants could choose the option “Prefer not to say” if they did not want to share that information.

We sent invitations to participate in twelve email lists that gather taxonomists, namely: Algae-L, Conch-L, Coral-List, Entomo-L, EvolDir, Mammal-L, MolluscaList, PaleoNet, Porifera List, Shark-L, Seagrass-Forum, Taxacom. Other email lists representing further taxonomic expertise were explored, but we could not successfully send messages on them. The survey remained available online for four weeks, from October 7^th^ to November 3^rd^ 2019. Only the present authors had access to the respondents’ answers.

### Analyses

All analyses were carried out in R version 4.1.0 [15]. Model selection was carried out via backwards selection, dropping non-significant terms in every step. The real identity of the interviewees was maintained anonymous in all cases, when interviewee ID is used, it was simply a unique number ranging from 1 to “n” (corresponding to the n^th^ respondent).

#### Question 11

In Q11, respondents scored how hard they found it to publish each of the taxonomic types of study. Their responses were converted to ordered factors with 5 levels (1 to 5), with 1 being easy and 5 hard. We then used the scores in two types of analyses.

First, we tested if the type of study explained the variation in scoring. For example, if respondents found it easier to make a synonym or to describe a new species. This analysis could not handle missing values and thus required respondents to have scored all categories. Thus, we focused only on the two study categories with the most responses: describing new species and synonymizing species. We used cumulative link models with random effects. Models were fitted with the “clmm2” function from the R package “ordinal” [16]. We used the interview score as response variables and the study type (synonymize or describe new species) as a categorical explanatory variable, also including the interviewee identity as a random effect. To compute the likelihood functions, models were fitted with the adaptive Gauss-Hermite quadrature method, with 10 quadrature nodes, and *p*-values were obtained via likelihood ratio tests.

Then, we tested whether the variation in the score for each of the five types of nomenclatural acts (synonymizing subspecies, synonymizing species, elevating subspecies, demoting species, or describing new species) depended on the characteristics of the study system or the respondent. We have included all possible nomenclatural acts pertaining to the species level, including subspecies; this was done to ensure our survey covers all sub-fields within taxonomy, even though subspecies are not used in many of them, notably for most invertebrates.

For each type of nomenclatural act we fitted a cumulative link model with the “clm” function from the “ordinal” R package [16] testing separately the effect of a) ‘region’ where the interviewee works, b) the studied ‘taxon’ (vertebrates, invertebrates, plants and other) and whether it is ‘fossil’ or ‘recent’, c) the ‘gender’, ‘degree’ and ‘academic position’ of the interviewee, and d) how many ‘years since degree’ the respondent has, with 3 categories (‘up to 3’, ‘3 to 10’ and ‘more than 10’). Whenever possible we included all categories marked by the respondents, but due to the very low number of responses in certain categories, we had to make adjustments: for the region, we combined Middle East and Asia; and for the degree, we could only consider bachelor, masters, and PhD. The other categories had to be excluded, but this was only done when the term ‘degree’ was present in the model, and we used the full dataset in any model that did not contain the term ‘degree’. Finally, in the case of the study type ‘demoting species’, the overall low number of responses also required us to remove the categories ‘prefer not to say’ in gender and ‘citizen scientist’ in academic position.

#### Questions 12 and 13

In Q12 and Q13 respondents marked whether their previous nomenclatural acts have been contested. For the analyses, the responses were grouped into a binary variable (1 = yes, 0 = no). Due to problems on the survey page, some responses were not recorded correctly (with both a yes and a no for the same category) and had to be excluded from all analyses (Q12 n = 5, Q13 n = 12). Similarly, to Q11, we used the responses in two analyses (separately for Q12 and Q13).

First, we tested if the probability of answering yes was based on the type of the study (synonym, new species, etc.). We used generalized linear mixed effect models (function “glmer” from the “lme4” R package; [17]), with a logit-link and Binomial error distribution, including whether or not the respondent answered yes as a response variable and the study type (using all study types) as a categorical explanatory variable, again, also including the interviewee identity as a random effect. Model comparison was performed via a parametric bootstrap with 1000 simulations using the function “PBmodcomp” of the R package “pbkrtest” [18].

Then, we tested whether the probability of answering yes for each type of study depended on the characteristics of the study system or the respondent. For each study type we used a generalized linear model with logit-link and Binomial error distribution (function “glm” base R), including whether or not the respondent answered yes as a response variable and a) the studied ‘taxon’ (vertebrates, invertebrates, plants, and other) and whether it is ‘fossil or recent’, b) the ‘region’, ‘gender’, ‘degree’, ‘academic position’ of the interviewee or c) how many ‘years since degree’ the interviewee has, with 3 categories (‘up to 3’, ‘3 to 10’ and ‘more than 10’). Similar to Q11, not all responses could be included due to the low number of responses in a few categories. Thus, once more we combined Middle East and Asia and for ‘degree’, we could only consider Bachelor’s, Master’s, and PhD when the term ‘degree’ was present in the model.

#### Question 14

We used an online word cloud generator [19] to highlight the most common words and topics referred to in our open-ended Q14 and to help to guide our qualitative analysis of the answers. To create the word cloud, words such as pronouns and connectives were removed, typos and misspelled words were corrected, and English was standardized to United States spelling.

## Results

We had a total of 634 respondents, nearly half (~48%) of which requested to be kept informed of our results (Q15). For the statistical analyses of Q11, Q12 and Q13, not all respondents answered all questions and thus the sample sizes varied depending on the test. Statistics are reported at the point of exclusion of the term from the model in the backwards selection procedure; *p*-values and estimates are only given for significant terms. For a complete overview of the results, please refer to the tables in the Supplementary Material (S1 File).

### Demographics

The respondents can be divided into groups according to gender identity, the country where they currently work, and academic background. Around 70% of the respondents were male, circa 28% female, and circa 1% gender diverse (Q1). Around 70% of respondents work in Europe or North America (Q2; S1 File: S1 Table); the two countries with the most respondents were the USA and Germany. Combining the countries in main regions, we have the following proportions: North America ~32%, Europe ~36%, Latin America ~16%, Oceania ~8%, Asia ~5%, Africa ~1.5%, Middle East ~1.5%. This classification in regions was used in further analyses, but due to the low number of responses from the Middle East, they were included in the category Asia.

About half the respondents work in universities (Q3; S1 File: S2 Table), with circa 27% working in museums, and 14% in other research institutes. The most common positions/jobs of the respondents (Q4; S1 File: S2 Table) were professor or lecturer (~31.5%), researcher (~31%, including postdoctoral researchers), and curator (~10%). About 10% of the respondents in the professor/lecturer category also had duties as curator or collection manager. Over 80% of respondents hold a doctoral degree (Q5; S1 File: S2 Table). Around 55% of respondents acquired their highest title (Q6) at least eleven years ago, while circa 30% acquired it from three to ten years ago, and circa 15% acquired it less than three years ago.

Regarding taxonomic group of expertise (Q7; S3 Table in S1 File), most respondents are entomologists (~17%), malacologists (~15%), phycologist (~10%), botanists (~10%) and mammalogists (~5%). For further analyses, we simplified this classification according to the typical main areas of research: Invertebrates (~57%, including tunicates), Botany (~22.5%, including algae, photosynthetic protozoans, and fungi), Vertebrates (~16%), and “Other” (~4.5%, including non-photosynthetic protozoans, bacteria, viruses, and those few researchers who work across two or more of the former categories). The vast majority of our respondents (Q8) work solely on extant taxa (~75%), while ~9% work solely on fossils, and ~16% work on both.

### Taxonomy

In Q9, we inquired about the last 3 journals in which the respondents had published a taxonomical work. Even though we had space for three entries only, a few respondents included up to six journals; we discarded those extra entries for our analysis. Not all respondents have published more than one taxonomic paper (and seven respondents indicated that they had no publications), so we only had 1651 entries of journal titles, averaging ~2.6 journals indicated per respondent. The journals where most taxonomic works were published by our respondents were, unsurprisingly: Zootaxa (~13%), ZooKeys (~5%), and Phytotaxa (~2%) (for the other most commonly used journals, see S1 File: S4 Table).

Circa 6.5% of our respondents have published papers on all five categories of nomenclatural acts we presented (Q10), namely: synonymization of subspecies; synonymization of species; elevation of subspecies to species; demotion of species to subspecies; description of new species. Not a single respondent published on four of these categories; ~16.5% of respondents published on three categories; ~29% of respondents publish on two categories; and the remaining published on only one category. Overall, ~92% of the respondents described a new species; ~60.5% synonymized species-level taxa; ~26% synonymized subspecies-level taxa; ~31% elevated a subspecies-level taxon to species-level; ~10.5% demoted a species-level taxon to subspecies.

Following Q10, we asked the respondents about their perceived difficulty in publishing on these topics (Q11), and whether their nomenclatural acts were contested during (Q12) and/or after (Q13) peer-review. To present the results of Q11 to Q13, we excluded those respondents who had never published on the nomenclatural act of interest.

#### Question 11

In Q11 (S1 File: S5 Table), the respondents assigned a value on a scale from 1 (very easy) to 5 (very difficult), regarding their perceived difficulty to publish each nomenclatural act. Overall, ~51.5% and 58.5% of respondents perceive synonymizing, respectively, subspecies- and species-level taxa as very easy or easy, while ~23.5% and ~20.5% find it neutral. Circa 61.5% and 49.5% of respondents perceive, respectively, elevating a subspecies to species and demoting a species to subspecies as very easy or easy, while ~22% and ~27.5% find it neutral. Finally, ~60% of respondents find describing new species to be easy or very easy, while ~22.5% find it neutral.

As explained above, to test whether the type of study explained the variation in scoring, we focused only on the two study categories with the most responses (describing new species and synonymizing species), given that the analysis cannot handle missing values. We retained 401 interviewees (out of 634) after filtering respondents who did not answer both questions. There was a borderline non-significant effect of type of question on scoring (LRT = 3.74, *p*-value = 0.05, OR = 0.74 [95% CI = 0.54–1.01]), with the odds of finding hard to publish being 26% lower for new species. In this test the interviewee identity accounted for a significant portion of the variation (LRT = 276.35, *p*-value < 0.01, [95% CI = 3.00–4.08]). Thus, different people have a significantly different perceptions of the difficulties of publishing, which is further explored in the second analysis.

**Fig 1:**
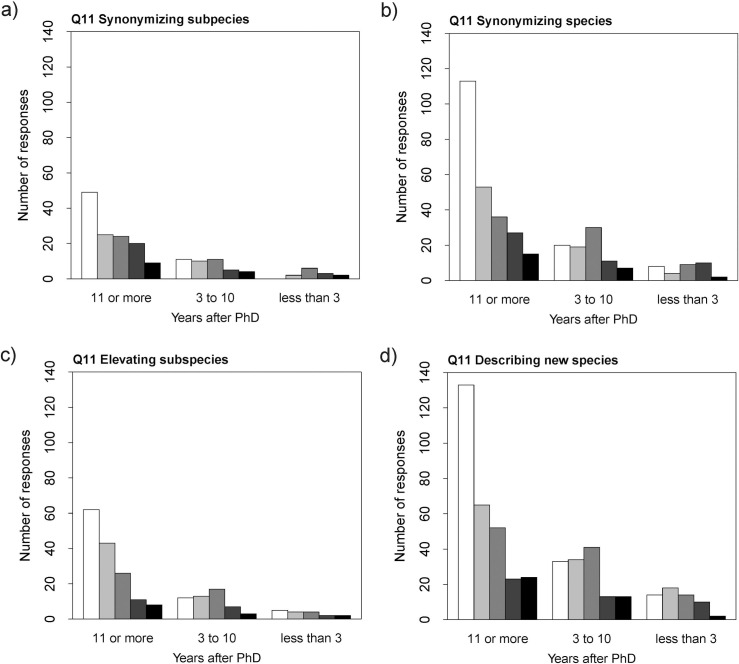
Responses to Q11. Number of responses of Q11 relative to years after PhD for different types of nomenclatural acts. Colours represent the scores from 1 to 5 increasing from lightest (1 = easiest) to darkest (5 = hardest).

The various questions differed in whether or not individual or study system characteristics explained the variation in responses (S1 File: S8 Table). One consistent individual characteristic was years since degree, which was a significant term in four out of five cases. Respondents further in their career consistently find it easier to publish studies than early-career researchers (Fig 1): synonymizing subspecies (χ2 = 7.76, *p-*value = 0.02; estimates relative to ‘11 or more’ years: ‘less than 3’ 1.33 ±0.49, ‘3 to 10’ 0.31 ±0.32); synonymizing species (χ2 = 19.79, *p-*value < 0.01; estimates relative to ‘11 or more’ years: ‘less than 3’ 1.09 ±0.33, ‘3 to 10’ 0.80 ±0.22); elevating subspecies (χ2 = 8.43, *p-*value = 0.01; estimates relative to ‘11 or more’ years: ‘less than 3’ 0.69 ±0.47, ‘3 to 10’ 0.78 ±0.29); describing new species (χ2 = 15.77, *p*-value < 0.01; estimates relative to ‘11 or more’ years: ‘less than 3’ 0.59 ±0.25, ‘3 to 10’ 0.69 ±0.).

**Fig 2:**
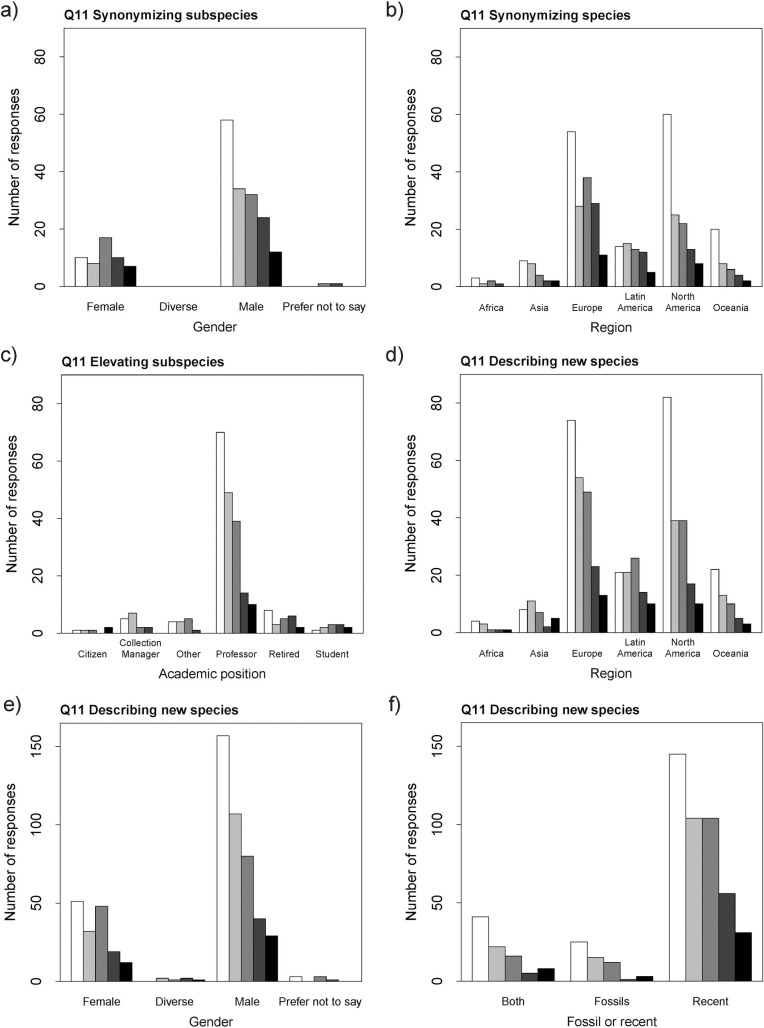
Responses to Q11. Number of responses of Q11 relative to various characteristics of the study system or respondent, for different types of nomenclatural acts. Colours represent the scores from 1 to 5 increasing from lightest (1 = easiest) to darkest (5 = hardest).

Gender was significant in two cases (Fig 2A and 2E), with a lower probability of male researchers finding it hard to synonymize subspecies or to describe new species: synonymizing subspecies (χ2 = 8.34, *p*-value = 0.02; estimates relative to ‘female’: prefer not to say -0.76 ±0.28 male: 0.61 ±1.10), describing new species (χ2 = 8.25, *p*-value = 0.04; estimates relative to ‘female’: diverse 1.15 ±0.71, male -0.35 ±0.17, prefer not to say -0.18 ±0.71).

Region was likewise significant in two cases (Fig 2B and 2D), with a lower probability of researchers in Oceania and North America finding it hard to synonymize species or to describe new species: synonymizing species (χ2 = 14.05, *p*-value = 0.02; estimates relative to ‘Africa’: Asia 0.06 ±0.77, Europe 0.42 ±0.69, Latin America 0.66 ±0,72, North America -0.15 ±0.70, Oceania -0.29 ±0.74), describing new species (χ2 = 16.03, *p*-value = 0.01; estimates relative to ‘Oceania’: Africa 0.54 ±0.66, Asia 0.21 ±0.59, Europe 0.77 ±0.61, Latin America -0.08 ±0.60, North America -0.04 ±0.63).

The academic position was significant only for elevating subspecies to species rank (Fig 2C), with the probability of finding it hard being higher for students (χ2 = 12.98, *p*-value = 0.02; estimates relative to ‘citizen scientist’: collection manager -1.56 ±1.02, other -1.31 ±1.03, professor -1.54 ±0.93, retired -0.83 ±1.00, student 0.08 ±1.06).

The nature of the study material was significant in one case (Fig 2F), in which the probability of finding hard to describe new species was reported lower for fossils (χ2 = 9.08, *p*-value = 0.01; estimates relative to ‘both’: fossil -0.12 ±0.31, recent 0.47 ±0.21).

#### Questions 12 and 13

**Fig 3:**
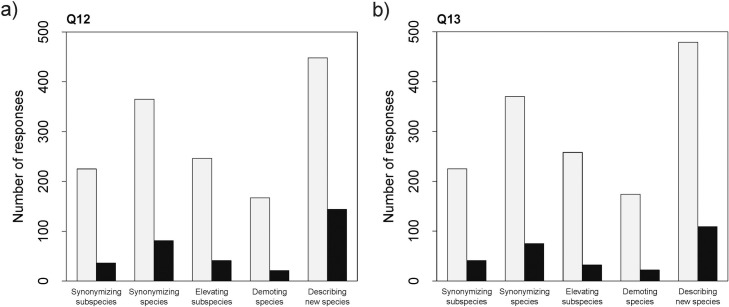
Responses to Q12 and Q13. Number of responses to Q12 and Q13 relative to the different types of study/ nomenclatural acts. Yes = black, no = light grey.

In Q12 (Fig 3A; S6 Table in S1 File), most of our respondents reported that their nomenclatural acts have never been contested during the peer-review process, either by reviewers or by editors. The nomenclatural acts were contested only ~14% of the time for synonymization of subspecies, ~18% for synonymization of species, ~14.5% for elevating subspecies to species level, ~11% for demoting species, and ~24.5% for describing new species. Most of the challenges to the acts in all categories happened in the past 5 years. The probability of having a nomenclatural act contested (yes = 1, no = 0) was significantly related to the type of the study (PBTest = 60.22, *p*-value <0.01), with describing new species being the most contested type (Fig 3A; S1 File: S9 Table).

**Fig 4:**
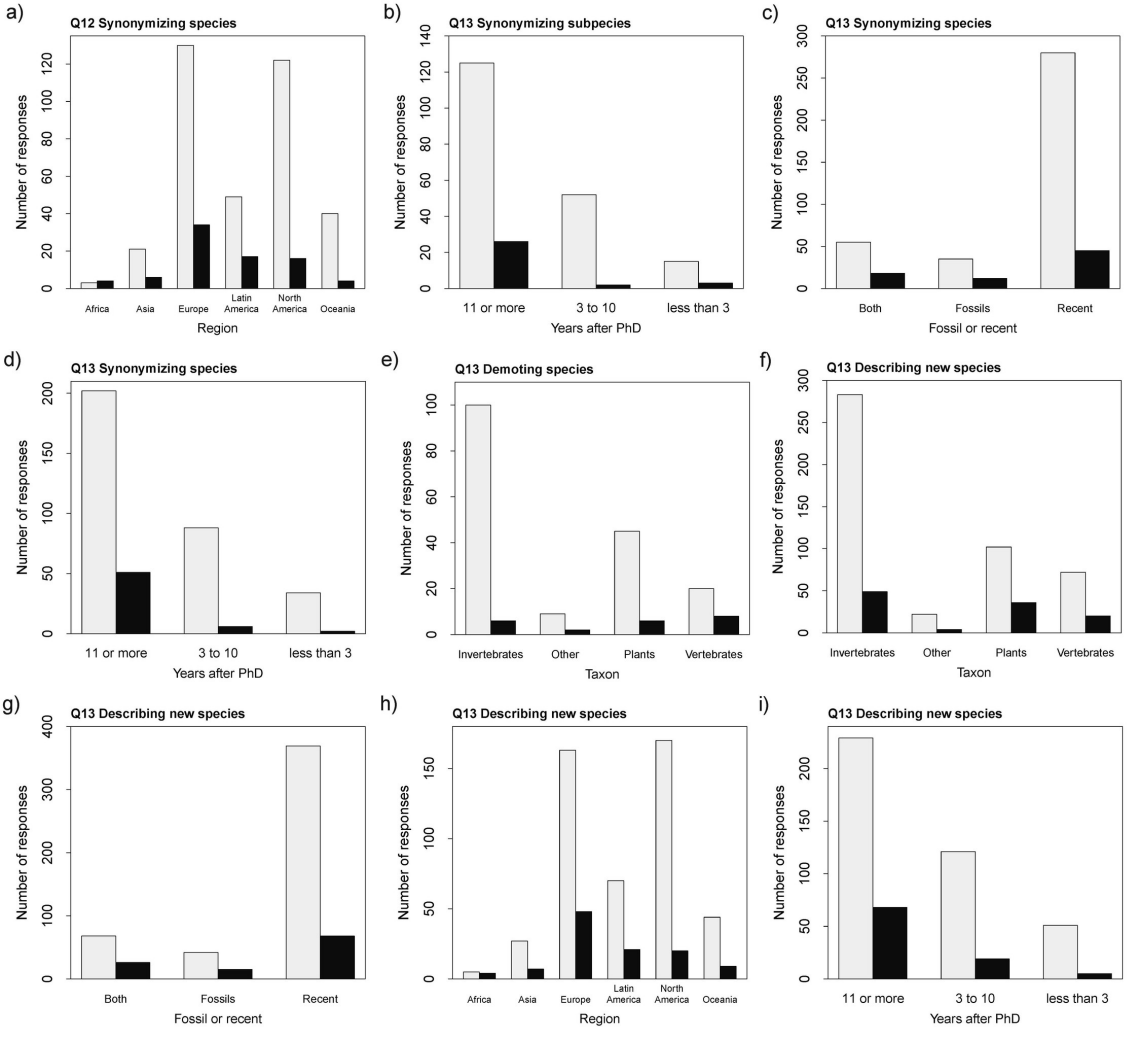
Responses to Q12 and Q13. Number of responses to Q12 and Q13 relative to various characteristics of the study system or respondent, for different types of nomenclatural acts. Yes = black, no = light grey.

Among the other analyses on Q12, only one test resulted in a significant value (S1 File: S10 Table). The probability of synonymization of species being contested during peer-review (Fig 4A) was higher for researchers based in Africa (χ2 = 15.96, *p*-value = 0.01; estimates: Africa 0.29 ±0.76, Asia -1.25 ±0.46, Europe -1.34 ±0.19, Latin America -1.06 ±0.28, North America -2.03 ±0.27, Oceania -2.30 ±0.52). This, however, might be an artifact due to the small number of respondents from that continent.

The overall numbers gathered from Q13 (Fig 3B; S1 File: S7 Table), concerning contesting of nomenclatural acts after peer-review, were overall very similar to those from Q12, except for new species descriptions, which were contested only in 19% of cases. The other nomenclatural acts were contested ~16.5% of the time for synonymization of subspecies, ~18.5% for synonymization of species, ~16% for elevating subspecies to species level, and ~10.5% for demoting species. Similar to what was observed for Q12, in Q13 the probability of being contested was significantly related to the type of the study (PBTest = 46.48, *p*-value <0.01), with describing new species being the most contested type (Fig 3B; S9 Table in S1 File).

**Fig 5:**
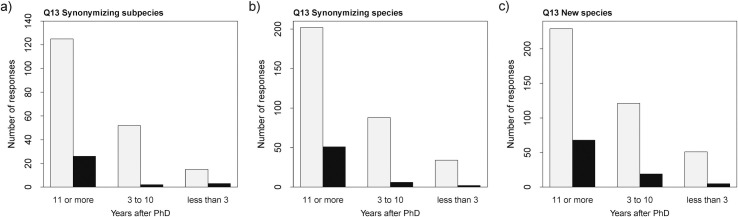
Responses to Q12 and Q13. Number of responses to Q12 and Q13 relative to years after PhD for different types of nomenclatural acts. Yes = black, no = light grey.

Among the responses to Q13, several terms were found to be significant (S1 File: S11 Table). Like in Q11, one consistent individual characteristic was years since degree, which was a significant term in three out of five cases (Fig 5). The probability of answering ‘yes’ is higher for early-career researchers for post-publication contestation of the following nomenclatural acts: synonymizing subspecies (χ2 = 7.77, *p*-value = 0.02; estimates: ‘less than 3’ -1.61 ±0.63, ‘3 to 10’ -3.26 ±0.72, ‘11 or more’ -1.57 ±0.22), synonymizing species (χ2 = 14.74, *p*-value < 0.01; estimates: ‘less than 3’ -2.83 ±0.73, ‘3 to 10’ -2.69 ±0.42, ‘11 or more’ -1.38 ±0.16), describing new species (χ2 = 10.07, *p*-value = 0.01; estimates: ‘less than 3’ -2.32 ±0.47, ‘3 to 10’ -1.85 ±0.25, ‘11 or more’ -1.21 ±0.14).

The study taxon was significant in two cases (Fig 4C and 4D). The probability of reporting a contested nomenclatural act post-publication was lower for invertebrates regarding the demotion of species to subspecies level (χ2 = 10.67, *p*-value = 0.01; estimates: ‘invertebrates’ -2.81 ±0.42, ‘other’ -1.50 ±0.78, ‘plants’ -2.01 ±0.43, ‘vertebrates’ -0.92 ±0.42), and lower for describing new species for the ‘other’ category (χ2 = 10.99, *p*-value = 0.01; estimates: ‘invertebrates’ -1.16 ±0.26, ‘other’ -1.38 ±0.56, ‘plants’ -0.37 ±0.30, ‘vertebrates’ -0.66 ±0.34).

The nature of the study material was significant in two cases (Fig 4B and 4E). The probability of reporting contestation of nomenclatural acts post-publication was lower for researchers working on living/recent species for the following acts: synonymizing species (χ2 = 7.32, *p*-value = 0.03; estimates: ‘both’ -1.12 ±0.27, ‘fossil’ -1.07 ±0.33, ‘recent’ -1.83 ±0.16), and describing new species (χ2 = 11.55, *p*-value < 0.01; estimates relative to ‘both’: ‘fossil’ -0.13 ±0.39, ‘recent’ -0.85 ±0.27).

Finally, region is significant in one case (Fig 4F). The probability of reporting contestation of new species descriptions post-publication is higher for researchers based in Africa (χ2 = 16.12, *p*-value = 0.01; estimates: ‘Africa’ -0.22 ±0.67, ‘Asia’ -1.35 ±0.42, ‘Europe’ -1.22 ±0.16, ‘Latin America’ -1.20 ±0.25, ‘North America’ -2.14 ±0.24, ‘Oceania’ -1.59 ±0.37).

#### Question 14

From the 634 respondents, 128 (~20%) left further comments in the answer to the non-mandatory Q14. Those included encouragement words, good wishes, suggestions, and even requests for topics for our eventual future surveys. A heartfelt thank you to all those respondents; it was truly great to read their comments. Naturally, there were also some comments more directly related to the matter at hand. We produced a word cloud with the answers to help to visualize the main points raised by the respondents (S1 File: S1 Fig). We describe and discuss those points further below. We quote small excerpts from the answers when pertinent, though the full answers are not reproduced here to maintain the respondents’ privacy.

## Discussion

Over six hundred respondents participated in our survey. Given that the number of taxonomists worldwide is deemed to be generally low [20–22], we consider this a sign that the community is cooperative and likes to have its voice heard. We have spotted significant results showing inequalities regarding gender, geographical region, and career stage. Differences regarding the nature of the specimens (fossil vs recent) and the study taxon were also detected. Finally, we observed a borderline effect regarding the description of new species being easier to publish than other nomenclatural acts. Concomitantly, new species descriptions are the most contested act both during peer-review and after publication.

### Gender

Around 70% of respondents identified themselves as males (Q1), demonstrating that gender balance remains at unacceptable levels in taxonomy. While it is recognized that the life sciences have a better representation of women than other areas in science and technology, that is mostly due to graduate students and postdoctoral researchers, while the gap in advanced career stages remains extreme, with only about 25% of full professors being female [23,24]. This was also reflected in our respondents: ~61% of the men acquired their highest title at least eleven years ago (Q6), while only ~41% of the women did so. Likewise, ~10% of the women were in the graduate student stage (Q5), while only ~6% of the men were in this stage.

We also identified a potential gender bias, since female researchers find it harder to publish nomenclatural acts (Q11; Fig 2A and 2E; S1 File: S8 Table). We consider this is not specific to taxonomy, but rather a more widespread problem, as gender bias in academic publishing has been recognized in other fields for a long time [e.g., 25,26].

### Geography

As expected, the vast majority (~68%) of respondents work in Europe or North America (Q2; S1 File: S1 Table). While the email lists that we used to disseminate the questionnaire are international in scope, they are entirely in English, which undoubtedly decreased our reach. Nevertheless, we had a relatively large number (~16%) of respondents from Latin America (notably Brazil, ranked third in the number of respondents; S1 File: S1 Table). It is recognized that developing countries are typically the most biodiverse, but lack the taxonomists, infrastructure, and funding to accomplish the monumental task of cataloguing their wildlife [4,27].

We detected some geographical bias (Q11; Fig 2B and 2D; S1 File: S8 Table), however, in which researchers in Oceania and North America consider it much easier to get their papers published than those in other continents. The language barrier could be playing a role here, as bias against non-English speakers is a recurring problem during the peer-review process [e.g., 28]. However, we cannot exclude other possibilities and it might prove fruitful to investigate this matter further. Another potential geographical bias is that researchers based in Africa (Q12, Q13; Fig 4A and 4F; S1 File: S10 and S11 Tables) showed a higher probability of their nomenclatural acts being contested both during peer-review and after publication, including the description of new species. However, the number of respondents from that continent was low and our results could be an artifact.

### Career stage

Most respondents work in universities or museums (Q3, S1 File: S2 Table: ~50% and 27%, respectively), with only circa 14% working in other research institutes. There is also a meaningful number of taxonomists working independently of academic institutions (~4%) and who do not have a degree beyond the Bachelor level (Q5, S1 File: S2 Table: ~6%). This is perhaps something particular to taxonomy, which is recognized as the only field in the biological sciences to count with significant input from “amateurs” [29,30], a fact that has been interpreted as both a boon and a curse by professional taxonomists [31]. Nevertheless, even the amateurs seem to be declining in number [20].

Over 70% of our respondents are researchers (including postdoctoral ones), professors/lecturers, or curators (Q4, S1 File: S2 Table). There is also a meaningful number (~8%) of retired professionals working in taxonomy, as expected [31]. The age bias is strong, with more than half of respondents being established/late-career researchers (Q4, Q6). Consequently, there is a dearth of graduate students (i.e., taxonomists in training) and early-career researchers working in taxonomy (Q4, S1 File: S2 Table: ~7.5% are graduate students; see also Q6: ~15% of respondents acquired their title less than three years ago), which is in line with reports in the literature signaling that the next generation of taxonomists is not being trained and that the field is slowly dying off, at least in developed countries [e.g., 6,22,32,33].

We detected a career stage bias in our responses: established/late-career researchers find it easier to publish their studies (Q11; Fig 1; S1 File: S8 Table), while early-career researchers have their published studies contested more often (Q13; Fig 5; S1 File: S11 Table). While it could be argued that this is a reflection of established researchers having more experience in publishing, the existence of a bias against early-career researchers has been reported in the literature [e.g., 34] and is also supported here by the respondents’ comments to Q14 (see below).

### Taxonomy

The taxonomic group of expertise (Q7, S1 File: S3 Table) of our respondents was in all likelihood biased by the selection of email lists that allowed us to post our message. For instance, while we got a good representation of most taxonomic groups, we received very few answers from mycologists, ornithologists, and dinosaur palaeontologists. Furthermore, two of the present authors are malacologists, which might have affected the large number of respondents specializing in molluscs (ranking second overall, with ~15%).

The journals most reported by the respondents as recent outlets for their publications (Q9) were well within expectations: Zootaxa, ZooKeys, and Phytotaxa. All three journals specialize in taxonomy and are supposed to be quick outlets for publications to alleviate the publishing part of the taxonomic impediment [35,36]. Zootaxa and Phytotaxa are free of charge for their authors (though not open access), but it was somewhat surprising to have ZooKeys listed in second place, given the journal’s hefty article processing charges. Other journals relatively frequently mentioned (S1 File: S4 Table) include area-specific ones such as Phycologia, Journal of Molluscan Studies, and Journal of Mammalogy, which could be expected by the number of respondents specializing in those taxa (Q7, S1 File: S3 Table).

The vast majority of our respondents (~92%) have published new species descriptions (Q10), while only ~60.5% and ~26% have synonymized, respectively, species-level or subspecies-level taxa. While there are undoubtedly many new species awaiting description, there is also a large amount of taxonomic “clean-up” to be done. So, could these numbers be an indication that there is a bias against “negative results” (i.e., synonymization)? In this scenario, authors and editors would prioritize publishing a new species description rather than revisions (where typically taxa end up being synonymized). That could be due to a host of reasons, but it is expected that most have to do with the ‘publish or perish’ culture (curriculum, impact, institutional requirements of publications, etc.), which affects most academic disciplines and not only taxonomy [e.g., 3,37]. Describing a new species is arguably much simpler than revising a whole set of established species in terms of work and amount of data involved: access to the material (including type specimens), visits to other natural history collections (typically located abroad), imaging, laboratory work, statistical analyses, etc. Finally, in most cases, there is an added glamour to describing a new species, especially given the urgency in bringing to light unknown species vis-à-vis the ongoing Sixth Extinction [38]. Meanwhile, taxonomic revisions tend to “clean up” the taxonomy of a given group and “reduce” its apparent biodiversity due to synonymization [39]; and there is a perceived diminishing number of revisionary works [3].

But is that bias experienced or at the very least perceived by working taxonomists? Our results could be pointing towards that scenario. According to the answers to Q11 (S1 File: S5 and S8 Tables), our respondents perceive the act of describing new species to be easier than synonymizing species (borderline *p*-value = 0.05). Furthermore, as delineated above, established/ late-career researchers and male researchers (particularly in Oceania and North America) find it easier to publish nomenclatural acts, in particular, new species descriptions (Figs 1 and 2; S1 File: S8 Table). Facets of gender, geographic, and career-stage biases were also perceived in some of the respondents’ answers to the open-ended Q14. We explore those in full detail further below, but it is worthwhile to note one particular response, a complaint that synonymizations are more difficult to get published if the author(s) of the species in question is(are) still alive.

At the same time, new species descriptions were reported as the most challenged nomenclatural acts during peer-review and after publication (Q12, Q13; Fig 3; S1 File: S9 Table). Contesting the content of a manuscript is partly how the quality of the final publication is insured by the peer-review system [40]. If species descriptions would have been contested due to more rigor being applied and more evidence being required, we would expect that the respondents consider this nomenclatural act difficult. We, however, observed the contrary: they consider the act of describing new species easier than other nomenclatural acts. As such, contestation of new species may be happening in response to subpar descriptions (though presently we cannot judge that).

However, there is an extra layer of complexity to our results: established/ late-career researchers consider publishing new species easy (Q11; Fig 1; S1 File: S8 Table), while early-career researchers have their new species contested more often (Q13; Fig 5; S1 File: S11 Table). It is recognized by taxonomists that the field can become very prone to personal opinions that overtake scientific criteria. It is also known that biased reviews amount to a significant fraction of the system [41–43]. We hope our initial impressions of the publication practices in taxonomy will lead to more investigation of the peer-review system in the field aiming toward equity and the eradication of biases.

### Respondents’ comments

The answers to our open-ended question (Q14; S1 File: S1 Fig) largely echo what is already widely recognized as the main general issues in taxonomy [7]: the unsatisfactory level of recognition the field has in academia; the lack of funding (especially in developing nations) for both research and for paying publishers’ charges (including open access fees); the difficulty in finding outlets to publish taxonomic works; and the inevitable low impact factor of those journals. Those issues are the sadly still mandatory “whining about the state of taxonomy” *sensu* [7]. But beyond those more immediate (and mostly external) concerns, we were also able to get glimpses of other (and internal) issues.

For instance, several respondents reported having experienced different degrees of bias and unethical behaviour in the peer-review system. The lack of accountability for reviewers (and the possibility of biased reviews) in the current peer-review system remains a major problem in academia [43–47] and taxonomy is no exception.

Some respondents also reported the difficulty for PhD candidates and early-career researchers to start a research line in taxonomy. This issue is in part linked to the peer-review problems above, but some respondents went further, singling out the issue that most taxonomists still operate as an “old-boys’ club” (direct quote), where certain older male researchers become authorities who cannot be challenged. Or, as put by one respondent: “In the fields to which I have been exposed, the ancient wise-one for a taxon still sits on all the panels/boards/chairs and stops younger/dissenting concepts getting a toe-hold. The idea of ‘peer’ is ridiculous in that context. The old guard considers themselves without peer (…).” It has been long recognized that the peer-review system has a bias against newcomers and “smaller” institutions in favour of a central elite in “top” universities [26,41], but very little has been actively done to correct this issue. Furthermore, the existence of an “ancient wise-one” in a field causes fewer papers to be published by external groups, though, in contrast, there’s a high influx of new papers and ideas soon after that person dies [48]. Given that there is a general recognition that the new generation of taxonomists is not being trained [22], this behaviour of taxonomists is a major concern, as it aggravates the issue and is counter-productive to the field. That is, the behaviour of some established/late-career taxonomists might be contributing to the collapse of taxonomy, which is a factor that has so far not been recognized among the many causes of the “taxonomic impediment”.

On a similar note, some respondents mentioned cases of “unsolicited peer-reviews” (direct quote). This refers to instances when their already peer-reviewed and published work is rejected by a committee of a given “official” checklist and thus becomes forever excluded from the mainstream handbooks and checklists. The committees, perhaps unsurprisingly, all belong to the vertebrate areas of study, with specific cases being mentioned by our respondents in herpetology, ichthyology, and ornithology. This ties in with the previous problem, given that typically such committees have biased demographics toward white male Anglophones [49].

There were also a few mentions of the problem of taxonomic vandalism, sometimes done with ill-intent and sometimes the result of the ill-advised or overzealous splitting of species due to minor and irrelevant morphological and/or genetic differences (see also [38] for a brief history of the so-called ‘mihi itch’). Malacology, in particular, was singled out by different respondents as a problematic area and fertile ground for taxonomic vandalism (see, for instance [50], for a review of a recent case). A further notable recent example comes from the field of herpetology [51]. Related to this problem, there was also mention of the lurking danger predatory and pay-to-publish journals might pose for taxonomy. Although a relatively recent phenomenon [52,53], the effects of predatory journals in taxonomy are already being felt in some areas [54].

## Conclusion

Taxonomy is the basis of all biological sciences and bad taxonomy can have large cascading impacts in other fields, from academic areas to nature conservation, public health, and even national economies [55–58]. Therefore, it is always helpful for taxonomists to analyse their field, recognize its problems, and work towards solving them.

Our results suggest that there is a potential bias against “negative results” (revisions and synonymization) in taxonomic publications, possibly abetted by the rush to describe species amid the current extinction crisis. Further gender, geographic and career-stage biases were also identified here. The present work represents only the first step, surveying taxonomists worldwide, and more focused questions and hypotheses need to be tested going forward.

In the meantime, however, we as a community should start thinking of measures to counteract such biases. Naturally, overhauling the whole ‘publish or perish’ culture would solve most problems, but that is of course not something taxonomists will achieve on their own or in the short term. Rather, we need to start thinking on smaller-scale and more immediately applicable measures that we can take, such as: working towards increased inclusiveness and equity; improving the definition of journal scopes and reviewer guidelines (including how to deal with unconscious bias); better informing and training editors to detect bias; being stricter with new species descriptions, requesting as much data as necessary and feasible (considering the available infrastructure in the most biodiverse countries); and, of course, giving more value and recognition to taxonomic revisions. Naturally, the latter must be extended beyond taxonomy’s inner circle to institutional committees and funding agencies.

Our respondents also highlighted (in the open-ended Q14) some potential avenues for future studies, some more directly related to the questions we investigated here, others only tangentially so but equally deserving of attention. These include: enquiring about the motivation for the new generation to pursue taxonomic studies and the hardships they encounter along the way; understanding how a taxonomist chooses (or ends up with) a given journal for their publications; investigating how referees deal with manuscripts they receive for peer-review and how to train/advise them for doing it better.

## Supporting information

S1 File Supporting information. Tables and figures with additional data on the survey responses and results of statistical analyses.
(PDF)

S1 Appendix (DOCX)

## References

[1] Ebach MC, Valdecasas AG, Wheeler QD. Impediments to taxonomy and users of taxonomy: accessibility and impact evaluation. Cladistics 2011;27: 550–557. doi: 10.1111/j.1096-0031.2011.00348.x 34875802

[2] Werner YL. The case of impact factor versus taxonomy: a proposal. Journal of Natural History 2006;40: 1285–1286.

[3] Guerra-García JM, Espinosa F, García-Gómez JC. Trends in Taxonomy today: an overview about the main topics in Taxonomy. Zoologica Baetica 2008;19: 15–49.

[4] Paknia O, Rajaei HS, Koch A. Lack of well-maintained natural history collections and taxonomists in megadiverse developing countries hampers global biodiversity exploration. Organisms Diversity & Evolution 2015;15: 619–629.

[5] Borkent A. Shrinking biodiversity, dwindling taxonomy and building a broader science. Megataxa 2020;1: 53–58.

[6] Britz R, Hundsdörfer A, Fritz U. Funding, training, permits—the three big challenges of taxonomy. Megataxa 2020;1: 49–52.

[7] Wheeler QD. Taxonomic shock and awe. In: Wheeler QD, editor. The New Taxonomy. Boca Raton: CRC Press; 2008. pp. 211–226.

[8] Woolf PD. Pressure to publish and fraud in science. Annals of Internal Medicine 1986;104: 254–256. doi: 10.7326/0003-4819-104-2-254 3946955

[9] Neill US. Publish or perish, but at what cost? Journal of Clinical Investigation 2008;118: 2368–2368. doi: 10.1172/JCI36371 18596904 PMC2439458

[10] Fanelli D. Do pressures to publish increase scientists’ bias? An empirical support from US states data. PLoS ONE 2010;5: e10271. doi: 10.1371/journal.pone.0010271 20422014 PMC2858206

[11] Olson CM, Rennie D, Cook M, Dickersin K, Flanagin A, Hogan JW, et al. Publication bias in editorial decision making. JAMA 2002;287: 2825–2828. doi: 10.1001/jama.287.21.2825 12038924

[12] Sandercock P. Negative results: why do they need to be published? International Journal of Stroke 2011;7: 32–33. 10.1111/j.1747-4949.2011.00723.x 22188851

[13] Fanelli D. Negative results are disappearing from most disciplines and countries. Scientometrics 2012;90: 891–904.

[14] Nissen SB, Magidson T, Gross K, Bergstrom CT. Publication bias and the canonization of false facts. eLife 2016;5: e21451. doi: 10.7554/eLife.21451 27995896 PMC5173326

[15] R Core Team. 2021. R: A Language and Environment for Statistical Computing. Vienna: R Foundation for Statistical Computing.

[16] Christensen RHB. ordinal—Regression Models for Ordinal Data. R package version 2019.12–10; 2019 [cited 2021 Aug 6]. Available from: https://CRAN.R-project.org/package=ordinal.

[17] Bates D, Mächler M, Bolker B, Walker S. Fitting linear mixed-effects models using lme4. Journal of Statistical Software 2015;67: 1–48.

[18] Halekoh U, Højsgaard S. A Kenward-Roger approximation and parametric bootstrap methods for tests in linear mixed models—the R package pbkrtest. Journal of Statistical Software 2014;59: 1–30.26917999

[19] Davies J. World Cloud Generator; 2018 [cited 2020 Nov 16]. Available from: https://www.jasondavies.com/wordcloud/.

[20] Hopkins GW, Freckleton RP. Declines in the numbers of amateur and professional taxonomists: implications for conservation. Animal Conservation 2002;5: 245–249.

[21] Drew LW. Are we losing the science of taxonomy? BioScience 2011;61: 942–946.

[22] Joppa LN, Roberts DL, Pimm SL. The population ecology and social behaviour of taxonomists. Trends in Ecology and Evolution 2011;26: 551–553. doi: 10.1016/j.tree.2011.07.010 21862170

[23] Sheltzer JM, Smith JC. Elite male faculty in the life sciences employ fewer women. PNAS 2014;111: 10107–10112. doi: 10.1073/pnas.1403334111 24982167 PMC4104900

[24] Moss-Racusin CA, van der Toorn J, Dovidio JF, Brescoll VL, Graham MJ, Handelsman J. A “Scientific Diversity” Intervention to Reduce Gender Bias in a Sample of Life Scientists. CBE Life Sciences Education 2017;15: 15:ar29. 10.1187/cbe.15-09-0187 PMC5008876 27496360

[25] Tregenza T. Gender bias in the refereeing process? Trends in Ecology and Evolution 2002;17: 349–350.

[26] Ersoy F, Pate J. Invisible hurdles: gender and institutional bias in the publication process in economics. SSRN 2021;2021: 3870368.

[27] Wilson EO. The encyclopedia of life. Trends in Ecology and Evolution 2003;18: 77–80.

[28] Clavero M. “Awkward wording. Rephrase”: linguistic injustice in ecological journals. Trends in Ecology and Evolution 2010;25: 552–553. doi: 10.1016/j.tree.2010.07.001 20692065

[29] Bouchet P. Inventorying the molluscan diversity of the world: what is our rate of progress? Veliger 1997;40: 1–11.

[30] Disney RHL. Insect biodiversity and the demise of alpha taxonomy. Antenna 1999;23: 84–88.

[31] Fontaine B, van Achterberg K, Alonso-Zarazaga MA, Araujo R, Asche M, Aspöck H, et al. New species in the Old World: Europe as a frontier in biodiversity exploration, a test bed for 21st century taxonomy. PLoS ONE 2012;7: e36881. doi: 10.1371/journal.pone.0036881 22649502 PMC3359328

[32] Smith GF, Figueiredo E. Capacity building in taxonomy and systematics. Taxon 2009;58: 697–699.

[33] Pearson DL, Hamilton AL, Erwin TL. Recovery plan for the endangered taxonomy profession. BioScience 2011;61: 58–63.

[34] Baker B. The bio workforce is getting older. Does it matter? BioScience 2018;68: 152.

[35] Zhang Z-Q. Accelerating biodiversity descriptions and transforming taxonomic publishing: the first decade of Zootaxa. Zootaxa 2011;2896: 1–7.

[36] Zhang Z-Q. Phytotaxa ten years on—the success of the foremost journal in botanical and mycological taxonomy. Phytotaxa 2019;423: 1–9.

[37] Tijdink JK, Schipper K, Bouter LM, Pont PM, de Jonge J, Smulder YM. How do scientists perceive the current publication culture? A qualitative focus group interview study among Dutch biomedical researchers. BMJ Open 2016;6: e008681. doi: 10.1136/bmjopen-2015-008681 26888726 PMC4762115

[38] Evenhuis NL. The “Mihi itch”—a brief history. Zootaxa 2008;1890: 59–68.

[39] Rosenberg G. A new critical estimate of named species-level diversity of the Recent Mollusca. American Malacological Bulletin 2014;32: 308–322.

[40] Kelly J, Sadeghieh T, Adeli K. Peer review in scientific publications: benefits, critiques, & a survival guide. Electronic Journal of the International Federation of Clinical Chemistry and Laboratory Medicine 2014;25: 227–243. 27683470 PMC4975196

[41] Peters DP, Ceci SJ. Peer-review practices of psychological journals: the fate of published articles, submitted again. Behavioral and Brain Sciences 1982;5: 187–255.

[42] Wennerås C, Wold A. Nepotism and sexism in peer-review. Nature 1997;387: 341–343. doi: 10.1038/387341a0 9163412

[43] Resnik DB, Gutierrez-Ford C, Peddada S. Perceptions of ethical problems with scientific journal peer review: an exploratory study. Science and Engineering Ethics 2008;14: 305–310. doi: 10.1007/s11948-008-9059-4 18311477 PMC2642979

[44] Smith R. Problems with peer review and alternatives. British Medical Journal 1988;296: 774–777. doi: 10.1136/bmj.296.6624.774 3126969 PMC2545379

[45] Cooper ML. problems, pitfalls, and promise in the peer-review process: commentary on Trafimow & Rice (2009). Perspectives on Psychological Science 2009;4: 84–90. doi: 10.1111/j.1745-6924.2009.01109.x 26158838

[46] Teixeira da Silva JA, Dobránszki J. Problems with traditional science publishing and finding a wider niche for post-publication peer review. Accountability in Research 2014;22: 22–40. 10.1080/08989621.2014.899909 25275622

[47] Ross-Hellauer T. What is open peer review? A systematic review. F1000Research 2017;6: 588. doi: 10.12688/f1000research.11369.2 28580134 PMC5437951

[48] Azoulay P, Fons-Rosen C, Zivin JSG. Does science advance one funeral at a time? American Economic Review 2019;109: 2889–2920. doi: 10.1257/aer.20161574 31656315 PMC6814193

[49] Moody A. Faculty diversity: problems and solutions. New York: Routledge Falmer; 2004.

[50] Páll-Gergely B, Hunyadi A, Auffenberg K. Taxonomic vandalism in malacology: comments on molluscan taxa recently described by N. N. Thach and colleagues (2014–2019). Folia Malacologica 2020;28: 35–76.

[51] Wüster W, Thomson SA, O’shea M, Kaiser H. Confronting taxonomic vandalism in biology: conscientious community self-organization can preserve nomenclatural stability. Biological Journal of the Linnean Society 2021;133: 645–670.

[52] Kearney MH. Predatory publishing: what authors need to know. Research in Nursing & Health 2014;38: 1–3. doi: 10.1002/nur.21640 25545343

[53] Eriksson S, Helgesson G. The false academy: predatory publishing in science and bioethics. Medicine, Health Care and Philosophy 2017;20: 163–170. 27718131 10.1007/s11019-016-9740-3 PMC5487745

[54] Raghavan R, Dahanukar N, Knight JDM, Bijukumar A, Katwate U, Krishnakumar K, et al. Predatory journals and Indian ichthyology. Current Science 2014;107: 740–742.

[55] Isely D. The disappearance. Taxon 1972;21: 3–12.

[56] McNeely JA. The role of taxonomy in conserving biodiversity. Journal for Nature Conservation 2002;10: 145–153.

[57] Dubois A. The relationships between taxonomy and conservation biology in the century of extinctions. Comptes Rendus Biologies 2003;326: S9–S21. doi: 10.1016/s1631-0691(03)00022-2 14558444

[58] Bortolus A. Error cascades in the biological sciences: the unwanted consequences of using bad taxonomy in ecology. AMBIO 2008;37: 114–118. doi: 10.1579/0044-7447(2008)37[114:ecitbs]2.0.co;2 18488554

