# Supplementary material for: Publication practice in Taxonomy: Global inequalities and potential bias against negative results
Source: PLoS One. 2022 Jun 1;17(6):e0269246. doi: 10.1371/journal.pone.0269246 (PMC9159550; doi:10.1371/journal.pone.0269246)
Supplement: S1 File — Tables and figures with additional data on the survey responses and results of statistical analyses. (PDF) [file pone.0269246.s001.pdf]

SUPPLEMENTARY MATERIAL

The following is the supplementary material to the article "*Publication practice in taxonomy: global inequalities and potential bias against negative results*" by Rodrigo Brincalepe Salvador, Daniel Caracanhas Cavallari, Douglas Rands & Barbara Mizumo Tomotani.

A. Answers

Below are presented tables and figures compiling the relative proportion of the answers to some of the questions, as well as additional figures that complement the results discussed in the main text. Please note that the raw results (anonymized) can be acquired from the corresponding author upon request.

Table S1. Answers to Q2, regarding the country where the respondents currently work.

Country	Percentage	Country	Percentage	Country	Percentage
United States of America	28.2	Peru	0.9	Estonia	0.2
Germany	9.8	Colombia	0.8	Greece	0.2
Brazil	6.5	Denmark	0.8	Honduras	0.2
Australia	5.7	Hungary	0.6	Hong Kong	0.2
United Kingdom	3.9	Poland	0.6	Israel	0.2
Spain	3.6	China	0.5	Luxembourg	0.2
Canada	3.5	Costa Rica	0.5	Malta	0.2
Mexico	3.2	Czech Republic	0.5	New Caledonia	0.2
Italy	3.0	Iran	0.5	Nigeria	0.2
France	2.5	Ireland	0.5	Pakistan	0.2
Argentina	2.2	Portugal	0.5	Philippines	0.2
India	2.2	Switzerland	0.5	Singapore	0.2
Netherlands	2.2	Croatia	0.3	Thailand	0.2
New Zealand	2.2	Ecuador	0.3	Turkey	0.2
Norway	1.4	Finland	0.3	Ukraine	0.2
Austria	1.3	Indonesia	0.3	United Arab Emirates	0.2
Belgium	1.3	Russia	0.3	Uruguay	0.2
South Africa	1.3	Taiwan	0.3	Venezuela	0.2
Sweden	1.3	American Samoa	0.2	Vietnam	0.2
Chile	1.1	Cyprus	0.2	Virgin Islands	0.2
Japan	1.1	Egypt	0.2		

Table S2. Answers to Q3, Q4 and Q5 regarding the respondent's academic background, namely: the institution where the respondents currently work (Q3), their position or job title (Q4), and their highest degree (Q5).

Institution	Percentage	Position / job title	Percentage	Highest degree	Percentage
University	48.8	Professor / Lecturer	31.4	Doctoral degree	82.6
Museum	26.9	Researcher (incl. postdoc)	30.8	Master's degree	11.8
Research institute	13.6	Curator	10.1	Bachelor's degree	4.3
Independent	4.2	Retired	8.2	High school	0.8
Government	1.9	Grad student	7.3	Trade / technical / vocational training	0.2
Industry	0.9	Collection manager	3.5	Other	0.3
NGO	0.8	Volunteer / Citizen scientist	2.5		
Other	2.7	Museum / lab technician	1.4		
		Undergrad student	0.5		
		Other	4.4		

Table S3. Answers to Q7: respondents' taxonomic group of expertise.

Taxon	Percentage	Taxon	Percentage
Arthropoda: Hexapoda	16.8	Arthropoda: Chelicerat	3.0
Mollusca	14.7	Fungi	1.8
algae	9.9	Platyhelminthes	1.8
plants	9.9	Echinodermata	1.6
Vertebrata: Mammalia	5.1	Annelida	1.4
Vertebrata: reptiles	4.2	protozoans	1.3
Cnidaria	4.1	Arthropoda: others	1.1
Porifera	4.1	Vertebrata: Dinosauria	0.8
Crustacea	3.9	Nematoda & Nemertea	0.8
Vertebrata: Amphibia	3.8	Brachiopoda	0.7
Vertebrata: fishes	3.8	Others	5.4

Table S4. Answers to Q9, regarding the journals where the respondents' have published their three most recent taxonomic works. Listed below are only the journals with six or more mentions.

Journal	Percentage	Journal	Percentage
Zootaxa	13.2	Biodiversity Data Journal	0.5
ZooKeys	4.8	Coleopterists Bulletin	0.5
Phytotaxa	2.2	Copeia	0.5
Zoological Journal of the Linnean Society	2.1	Cretaceous Research	0.5
Molecular Phylogenetics and Evolution	1.9	Journal of Crustacean Biology	0.5
Taxon	1.7	Journal of Zoological Systematics and Evolutionary Research	0.5
Phycologia	1.6	PhytoKeys	0.5
Journal of Phycology	1.6	Proceedings of the Entomological Society of Washington	0.5
PLoS ONE	1.6	Insecta Mundi	0.4
European Journal of Taxonomy	1.4	International Journal of Plant Sciences	0.4
Journal of Paleontology	1.3	Journal of Parasitology	0.4
Journal of Molluscan Studies	1.1	Scientific Reports	0.4
Journal of the Marine Biological Association of the United Kingdom	1.0	Systematic Parasitology	0.4
PeerJ	1.0	Acta Entomologica Musei Nationalis Pragae	0.4
Marine Biodiversity	0.9	Acta Palaeontologica Polonica	0.4
Journal of Mammalogy	0.8	Alcheringa	0.4
European Journal of Phycology	0.8	Basteria	0.4
Systematic Entomology	0.8	Biological Journal of the Linnean Society	0.4
Zoologica Scripta	0.8	BMC Evolutionary Biology	0.4
The Nautilus	0.7	Diatom Research	0.4
Invertebrate Systematics	0.7	Malacologia	0.4
Journal of Natural History	0.7	Palaeontology	0.4
Journal of Vertebrate Paleontology	0.7	Plant Systematics and Evolution	0.4
Systematic Biology	0.7	Protist	0.4
Cladistics	0.6	Others	43.4

Table S5. Answers to Q11, regarding the difficulty level perceived by respondents to publish each type of nomenclatural act. To calculate the percentages, we excluded all respondents who never published a paper on the nomenclatural act in question.

Difficulty	Synonymizing subspecies	Synonymizing species	Elevating subspecies	Demoting species	Describing new species
1 (very easy)	31.8%	38.2%	35.3%	28.9%	35.9%
2 (easy)	19.6%	20.3%	26.2%	20.7%	24.0%
3 (neutral)	23.4%	20.3%	21.8%	27.4%	22.4%
4 (hard)	16.4%	14.6%	10.3%	11.9%	10.5%
5 (very hard)	8.9%	6.7%	6.3%	11.1%	7.1%

Table S6. Answers to Q12, regarding whether the respondents' nomenclatural acts have been contested during the peer-review process, by either reviewers and/or editors. To calculate the percentages, we excluded all respondents who never published a paper on the nomenclatural act in question.

Has the nomenclatural act been contested?	Synonymizing subspecies	Synonymizing species	Elevating subspecies	Demoting species	Describing new species
No	86.2%	81.8%	85.4%	88.8%	75.5%
Yes, in the past 5 years	8.4%	11.2%	9.7%	5.9%	16.0%
Yes, more than 5 years ago	2.7%	4.5%	1.7%	2.7%	6.6%
Yes, but I don't remember when	2.7%	2.5%	3.1%	2.7%	1.9%

Table S7. Answers to Q13, regarding whether the respondents' nomenclatural acts have been contested after publication (for instance, by other academic articles or "official" checklists). To calculate the percentages, we excluded all respondents who never published a paper on the nomenclatural act in question.

Has the nomenclatural act been contested?	Synonymizing subspecies	Synonymizing species	Elevating subspecies	Demoting species	Describing new species
No	84.3%	81.5%	87.8%	89.5%	80.9%
Yes, in the past 5 years	8.6%	9.3%	8.0%	7.2%	9.8%
Yes, more than 5 years ago	3.4%	5.6%	2.3%	1.1%	6.4%
Yes, but I don't remember when	3.7%	3.6%	1.9%	2.2%	2.9%

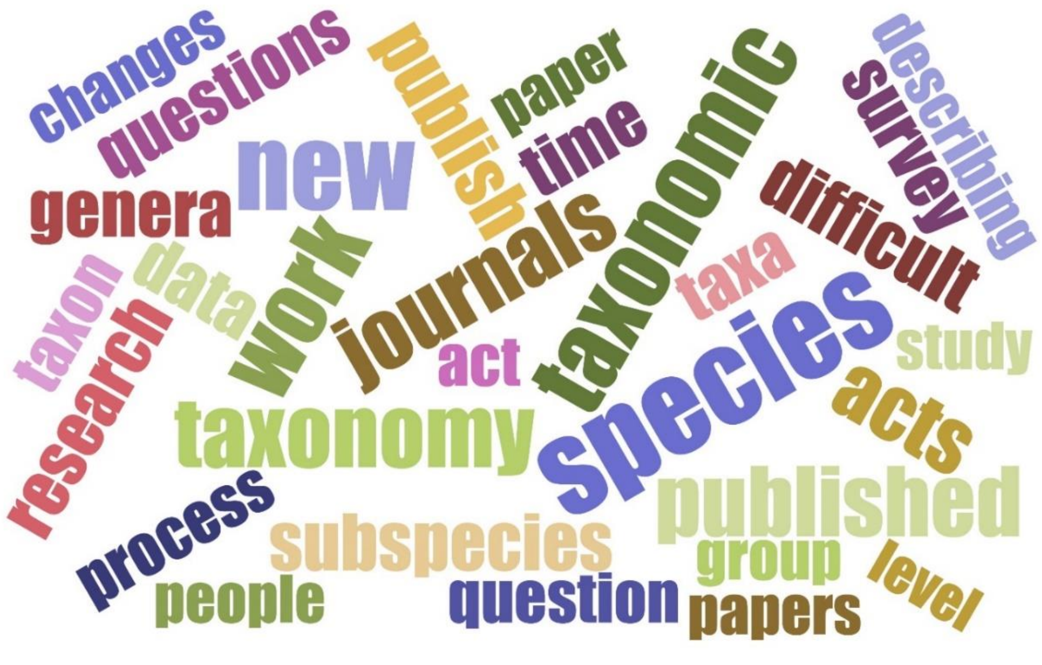

Figure S1. Tag cloud of keywords from the respondents' answers to Q14. The full answers are not reproduced here to maintain privacy.

B. Statistical Analyses

Below are presented the tables pertaining to the statistical analyses performed for Q11, Q12, and Q13 (see Material & Methods).

Table S8. Results from the tests pertaining to Q11 (reported difficulty in publishing different nomenclatural acts).

**Synonymizing subspecies (n=214)**	**Estimate**	**s.e.**	**χ2**	***p*-value**
a) Region			5,56	0,35
b) Taxa			1,60	0,66
b) Fossil or recent			3,87	0,21
c) Gender (female)			8,34	**0,02**
c) Gender (male)	-0,76	0,28		
c) Gender (prefer not to say)	0,61	1,10		
c) Degree			0,24	0,89
c) Academic position			5,15	0,40
d) Years since degree (less than 3)	1,33	0,49	7,76	**0,02**
d) Years since degree (3 to 10)	0,31	0,32		
d) Years since degree (11 or more)				

**Synonymizing species (n=419)**	**Estimate**	**s.e.**	**χ2**	***p*-value**
a) Region (Africa)			14,05	**0,02**
a) Region (Asia)	0,06	0,77		
a) Region (Europe)	0,42	0,69		
a) Region (Latin America)	0,66	0,72		
a) Region (North America)	-0,15	0,70		
a) Region (Oceania)	-0,29	0,74		
b) Taxa			2,29	0,51
b) Fossil or recent			2,42	0,30
c) Gender			7,56	0,06
c) Degree			0,02	0,99
c) Academic position			8,50	0,13
d) Years since degree (less than 3)	1,09	0,33	19,79	**< 0.01**
d) Years since degree (3 to 10)	0,80	0,22		
d) Years since degree (11 or more)				

**Elevating subspecies (n=252)**	**Estimate**	**s.e.**	**χ2**	***p*-value**
a) Region			2,81	0,73
b) Taxa			1,78	0,62
b) Fossil or recent			2,43	0,30
c) Gender			1,65	0,20
c) Degree			5,09	0,08
c) Academic position (Citizen scientist)			12,98	**0,02**
c) Academic position (Collection manager)	-1,56	1,02		
c) Academic position (Other)	-1,31	1,03		
c) Academic position (Professor)	-1,54	0,93		
c) Academic position (Retired)	-0,83	1,00		
c) Academic position (Student)	0,08	1,06		
d) Years since degree (less than 3)	0,69	0,47	8,43	**0,01**
d) Years since degree (3 to 10)	0,78	0,29		
d) Years since degree (11 or more)				

**Demoting species (sample = 135)**	**Estimate**	**s.e.**	**χ2**	***p*-value**
a) Region			6,85	0,23
b) Taxa			3,82	0,28
b) Fossil or recent			2,76	0,25
c) Gender			1,74	0,19
c) Degree			2,48	0,29
c) Academic position			2,19	0,70
d) Years since degree			5,74	0,06

**Describing new species (sample = 588)**	**Estimate**	**s.e.**	**χ2**	***p*-value**
a) Region (Africa)	0,54	0,66	16,03	**0,01**
a) Region (Asia)	0,21	0,59		
a) Region (Europe)	0,77	0,61		
a) Region (Latin America)	-0,08	0,60		
a) Region (North America)	-0,04	0,63		
a) Region (Oceania)				
b) Taxa			4,18	0,24
b) Fossil or recent (both)			9,08	**0,01**
b) Fossil or recent (fossil)	-0,12	0,31		
b) Fossil or recent (recent)	0,47	0,21		
c) Gender (female)			8,25	**0,04**
c) Gender (diverse)	1,15	0,71		
c) Gender (male)	-0,35	0,17		
c) Gender (prefer not to say)	-0,18	0,71		
c) Degree			1,52	0,47
c) Academic position			4,58	0,47
d) Years since degree (less than 3)	0,59	0,25	15,77	**< 0.01**
d) Years since degree (3 to 10)	0,69	0,19		
d) Years since degree (11 or more)				

Table S9. Probability of nomenclatural acts being contested during peer-review (Q12) and after publication (Q13).

**Q12: probability of being contested**	**Estimate**	**s.e.**	**PBTest**	***p*-value**
Type			60,22	**< 0.01**
Synonymization subspecies	-8,48	0,67		
Synonymization species	-7,52	0,58		
Elevate subspecies	-8,45	0,65		
Demote species	-9,07	0,75		
New species	-6,36	0,48		
**Q13: probability of being contested**	**Estimate**	**s.e.**	**PBTest**	***p*-value**
Type			46,48	**< 0.01**
Synonymization subspecies	-7,70	0,62		
Synonymization species	-6,82	0,53		
Elevate subspecies	-8,54	0,65		
Demote species	-8,85	0,71		
New species	-6,64	0,50		

Table S10. Results from the tests pertaining to Q12 (contestation to nomenclatural acts during peer-review).

**Synonymizing subspecies (n=262)**	**Estimate**	**s.e.**	**χ2**	***p*-value**
a) Taxa			5,22	0,16
a) Fossil or recent			0,28	0,87
b) Region			8,54	0,13
b) Gender			1,06	0,79
b) Degree			1,10	0,58
b) Academic position			2,79	0,73
c) Years since degree			3,95	0,14
**Synonymizing species (n=447)**	**Estimate**	**s.e.**	**χ2**	***p*-value**
a) Taxa			2,14	0,54
a) Fossil or recent			1,06	0,59
b) Region (Africa)	0,29	0,76	15,96	**0,01**
b) Region (Asia)	-1,25	0,46		
b) Region (Europe)	-1,34	0,19		
b) Region (Latin America)	-1,06	0,28		
b) Region (North America)	-2,03	0,27		
b) Region (Oceania)	-2,30	0,52		
b) Gender			5,37	0,15
b) Degree			0,11	0,94
b) Academic position			3,15	0,68
c) Years since degree			1,20	0,55

**Elevate subspecies (n=288)**	**Estimate**	**s.e.**	**χ2**	***p*-value**
a) Taxa			7,37	0,06
a) Fossil or recent			0,29	0,86
b) Region			5,22	0,39
b) Gender			0,99	0,80
b) Degree			0,63	0,73
b) Academic position			6,53	0,26
c) Years since degree			4,27	0,12
**Demote species (sample = 188)**	**Estimate**	**s.e.**	**χ2**	***p*-value**
a) Taxa			4,55	0,21
a) Fossil or recent			3,96	0,14
b) Region			8,22	0,14
b) Gender			0,85	0,84
b) Degree			0,43	0,81
b) Academic position			8,23	0,14
c) Years since degree			5,28	0,07
**New species (sample = 496)**	**Estimate**	**s.e.**	**χ2**	***p*-value**
a) Taxa			3,20	0,36
a) Fossil or recent			0,50	0,78
b) Region			10,31	0,07
b) Gender			1,30	0,73
b) Degree			3,33	0,19
b) Academic position			2,22	0,82
c) Years since degree			2,44	0,30

Table S11. Results from the tests pertaining to Q13 (contestation to nomenclatural acts after publication).

**Synonymization subspecies (n=267)**	**Estimate**	**s.e.**	**χ^2**	***p*-value**
a) Taxa			2,99	0,39
a) Fossil or recent			2,49	0,29
b) Region			4,18	0,52
b) Gender			4,52	0,21
b) Degree			0,39	0,82
b) Academic position			1,80	0,88
d) Years since degree (less than 3)	-1,61	0,63	7,77	**0,02**
d) Years since degree (3 to 10)	-3,26	0,72		
d) Years since degree (11 or more)	-1,57	0,22		

**Synonymization species (n=448)**	**Estimate**	**s.e.**	**χ^2**	***p*-value**
a) Taxa			4,06	0,25
a) Fossil or recent (both)	-1,12	0,27	7,32	**0,03**
a) Fossil or recent (fossil)	-1,07	0,33		
a) Fossil or recent (recent)	-1,83	0,16		
b) Region			10,69	0,06
b) Gender			5,98	0,11
b) Degree			2,01	0,37
b) Academic position			5,42	0,37
d) Years since degree (less than 3)	-2,83	0,73	14,74	**<0.01**
d) Years since degree (3 to 10)	-2,69	0,42		
d) Years since degree (11 or more)	-1,38	0,16		

**Elevate subspecies (n=293)**	**Estimate**	**s.e.**	**χ^2**	***p*-value**
a) Taxa			3,24	0,36
a) Fossil or recent			3,13	0,21
b) Region			6,04	0,30
b) Gender			4,44	0,22
b) Degree			0,85	0,65
b) Academic position			6,73	0,24
c) Years since degree			4,24	0,12

**Demote species (sample = 199)**	**Estimate**	**s.e.**	**χ^2**	***p*-value**
a) Taxa (invertebrates)	-2,81	0,42	10,67	**0,01**
a) Taxa (other)	-1,50	0,78		
a) Taxa (plants)	-2,01	0,43		
a) Taxa (vertebrates)	-0,92	0,42		
a) Fossil or recent			0,47	0,79
b) Region			9,95	0,08
b) Gender			3,01	0,39
b) Degree			0,51	0,78
b) Academic position			10,70	0,06
c) Years since degree			1,65	0,44

**New species (sample = 594)**	**Estimate**	**s.e.**	**χ^2**	***p*-value**
a) Taxa (invertebrates)	-1,16	0,26	10,99	**0,01**
a) Taxa (other)	-1,38	0,56		
a) Taxa (plants)	-0,37	0,30		
a) Taxa (vertebrates)	-0,66	0,34		
a) Fossil or recent (fossil)	-0,13	0,39	11,55	**< 0.01**
a) Fossil or recent (recent)	-0,85	0,27		
b) Region (Africa)	-0,22	0,67	16,12	**0,01**
b) Region (Asia)	-1,35	0,42		
b) Region (Europe)	-1,22	0,16		
b) Region (Latin America)	-1,20	0,25		
b) Region (North America)	-2,14	0,24		
b) Region (Oceania)	-1,59	0,37		
b) Gender			2,99	0,39
b) Degree			3,11	0,21
b) Academic position			2,05	0,84
d) Years since degree (less than 3)	-2,32	0,47	10,07	**0,01**
d) Years since degree (3 to 10)	-1,85	0,25		
d) Years since degree (11 or more)	-1,21	0,14		
