# Supplementary material for: Publication practice in Taxonomy: Global inequalities and potential bias against negative results
Source: PLoS One. 2022 Jun 1;17(6):e0269246. doi: 10.1371/journal.pone.0269246 (PMC9159550; doi:10.1371/journal.pone.0269246)
Supplement: S1 Appendix — (DOCX) [file pone.0269246.s002.docx]

# **Appendix**

The survey is fully reproduced below, although differing in appearance from the version available to the respondents on Google Forms (Google Inc.). Questions marked with an asterisk (*) were mandatory.

**Publication trends in taxonomy**

Dear fellow taxonomist,

We are investigating the publication culture in taxonomy across all taxa. The aim of the present questionnaire is to assess some trends in current taxonomic publications and its peer-review process. The data gathered will be used for publication on an academic journal.

The questionnaire should take no more than 5 minutes of your time. It is entirely anonymous and only the researchers listed below will have access to the data. However, at the end of the questionnaire, you can opt to leave your email address to receive updates of this project.

Thank you very much for your cooperation!

Kind regards,

Salvador, Cavallari & Tomotani.

**1.** Gender identity *

- Female
- Male
- Gender diverse
- Prefer not to say

**2.** In which country do you currently work? *

______________________________

**3.** In what kind of institution do you currently work? *

- University
- Museum
- Research institute
- NGO
- Industry
- Other: __________

**4.** What is your current position/ job title? *

- Undergrad student
- Grad student
- Researcher (including postdoc)
- Professor / Lecturer
- Curator
- Collection manager
- Museum/Lab technician
- Retired
- Volunteer / Citizen scientist
- Other: __________

**5.** Which is your highest degree? *

- Doctoral degree
- Master's degree
- Bachelor's degree
- Trade/ technical/ vocational training
- High school
- Other: __________

**6.** How many years have elapsed since you achieved your highest degree? *

- Less than 3 years
- 3 to 10 years
- 11 years or more

**7.** Which taxon/taxa do you specialize in? (Ex: Hexapoda, Mollusca, birds, flowering plants) *

______________________________

**8.** With which kind of specimens do you work with? *

- Living / Recent (including archaeological and sub-fossil specimens)
- Fossils
- Both

**9.** Please provide the name of the last 3 journals in which you published a taxonomic work. *

______________________________

______________________________

______________________________

**10.** What kinds of nomenclatural acts you already published on? (Check all that apply.) *

- Synonymization of two (or more) subspecies (including forms, varieties, etc.)
- Synonymization of two (or more) species
- Elevating subspecies to species level
- Demoting species to subspecies level
- New species description

**11.** On a scale of 1 to 5, how difficult you find to publish each of the following nomenclatural acts? (1 is very easy; 5 is very difficult) *

**12.** Were any of your nomenclatural acts contested during the peer-review process, by reviewers and/or editors? *

**13.** Were any of your nomenclatural acts contested after publication? This includes other academic articles and “official” lists (e.g., World Bird List, WoRMS). *

**14.** If you wish, please feel free to leave any additional comments here:

______________________________

**15.** If you'd like to hear about our results, please provide your email address below:

______________________________
